# Correction to: Setting an evolutionary trap: could the hider strategy be maladaptive for white-tailed deer?

**DOI:** 10.1007/s10164-017-0536-6

**Published:** 2017-12-01

**Authors:** M. Colter Chitwood, Marcus A. Lashley, Christopher E. Moorman, Christopher S. DePerno

**Affiliations:** 1 Fisheries and Wildlife Sciences Department, University of Missouri, 302 Anheuser-Busch Natural Resources Building, Columbia, MO 65211 USA; 2 Department of Wildlife, Fisheries, and Aquaculture, Mississippi State University, Mississippi State, MS 39762 USA; 3 Fisheries, Wildlife, and Conservation Biology Program, Department of Forestry and Environmental Resources, North Carolina State University, Raleigh, NC 27695 USA; 4 Present Address: Wildlife Biology Program, Department of Ecosystem and Conservation Sciences, University of Montana, 32 Campus Drive, Missoula, MT 59812 USA

## Correction to: J Ethol (2017) 35:251–257 10.1007/s10164-017-0514-z

The article, “Setting an evolutionary trap: could the hider strategy be maladaptive for white-tailed deer?”, written by M. Colter Chitwood, Marcus A. Lashley, Christopher E. Moorman and Christopher S. DePerno, was originally published Online First without open access. After publication in volume 35, issue 3, pages 251–257 the author decided to opt for Open Choice and to make the article an open access publication. Therefore, the copyright of the article has been changed to © The Author(s) 2018 and the article is forthwith distributed under the terms of the Creative Commons Attribution 4.0 International License (http://creativecommons.org/licenses/by/4.0/), which permits use, duplication, adaptation, distribution and reproduction in any medium or format, as long as you give appropriate credit to the original author(s) and the source, provide a link to the Creative Commons license, and indicate if changes were made.

